# Elucidation of Underlying Mechanisms by Which *Millettia macrophylla* Benth Induces Its Estrogenic Activity

**DOI:** 10.1155/2014/763781

**Published:** 2014-08-10

**Authors:** Stéphane Zingue, Chantal Beatrice Magne Nde, Colin Clyne, Dieudonné Njamen

**Affiliations:** ^1^Laboratory of Physiology, Department of Life and Earth Sciences, Higher Teachers' Training College, University of Maroua, P.O. Box 55, Maroua, Cameroon; ^2^Laboratory of Animal Physiology, Department of Animal Biology and Physiology, Faculty of Science, University of Yaounde I, P.O. Box 812, Yaounde, Cameroon; ^3^MIMR-PHI Institute of Medical Research, Departments of Molecular and Translational Sciences, Monash University, 246 Clayton Road, Level 4, VIC 3168, Melbourne, Australia

## Abstract

*Millettia macrophylla* is used traditionally to treat menopause related symptoms. This plant was shown to exhibit estrogenic effects *in vitro* on human embryonic kidney cells and *in vivo* on ovariectomized rats. The present study aimed at elucidating underlying mechanisms by which *M. macrophylla* induced its estrogenic effects. To accomplish our goal, kidney Hek293T cells transiently transfected with estrogen alpha or beta receptor expression plasmids were cotreated with a pure antiestrogen ICI 182,780 and the dichloromethane or methanol soluble fractions of *M. macrophylla.* To follow up, we cotreated ovariectomized rats with both extracts and ICI 182,780 for 3 days in the classical uterotrophic assay. Animals were then sacrificed and the uterine wet weight, total protein levels in uteri, uterine, and vaginal epithelial heights, and mammary gland were assessed. *In vitro,* the results suggested that the induction of the estrogenic activity by *M. macrophylla* is due to the binding of its secondary metabolites to ER*α* and ER*β*. *In vivo*, the cotreatment of extracts and ICI 182,780 significantly abrogated the biological responses induced by the extracts alone. Taken together, these results indicate that the active principles of *M. macrophylla* induce their beneficial effects on menopausal symptoms by activating the ERs.

## 1. Introduction

Throughout the ages, plants have been the major source of medication for the treatment of diverse diseases and continue to be a major source of primary health care for today's populations in most areas of the world [1]. The medicinal value of plants has therefore been recognized by almost every society on this planet [2]. In the developing countries in particular, 80% of the population still resort to traditional medicine for their primary health care [3]. For instance, the use of traditional herbal medicine is popular in Central Africa, particularly in Cameroon, mainly because of economic constraints and the strength of traditional beliefs [4, 5]. In our ongoing search for bioactive compounds from medicinal plants [6–10], we have been studying the stem bark of* Millettia macrophylla,* a Cameroonian medicinal plant used in the treatment of menopausal symptoms.

*Millettia macrophylla,* also known as* Millettia aboensis,* is used to alleviate sexual transmitted diseases, as well as some symptoms related to menopausal complaints, like other species of Millettia genus such as* M. conraui*,* M. drastica*,* M. griffoniana*,* M. zechiana*,* M. barteri,* and* M. dura* [11–17]. Since there were no scientific data to support the above claims, we therefore evaluated the beneficial effects of* M. macrophylla* extracts on menopausal symptoms, in order to scientifically justify the claimed beneficial effects. Using a model of rats exhibiting menopause-like symptoms* in vivo* and cells overexpressing estrogen receptors alpha or beta (ER*α* or ER*β*)* in vitro*, we previously showed that DCM and MeOH extracts of* M. macrophylla* mimic estrogen-like effects on the genital tract and increase estrogen receptors transactivity, respectively [18]. These results suggest that* M. macrophylla* may contain secondary metabolites that bind ERs or alter coregulators of ERs. However, the mechanism of actions that underpin the aforementioned effects is still unknown. Therefore, we aimed to elucidate the underlying mechanisms by which* M. macrophylla* induces its estrogenic effects. To accomplish our goal, DCM and MeOH soluble fractions of* M. macrophylla* were used with a pure and well-characterized antiestrogen ICI 182,780 in a cotreatment regimen* in vitro* on cells transiently transfected with the estrogen *α* or *β* receptor expression plasmids and* in vivo* in ovariectomized rats.

## 2. Material and Methods

### 2.1. Substances

The pure antiestrogen ICI 182,780 (7-[9-[4,4,5,5,5-pentafluoropentyl) sulfinyl [nonyl]-estra-1,3,5(10)-triene-3,17diol) and 17*β*-3-benzoate (E2B) (Estr-1, 3,5 (10)-trien-3, 16*α*, 17*β*-triol) were obtained from Sigma-Aldrich (Hamburg, Germany). The HEK293T cells that contain the SV40 large T-antigen were purchased from ATCC (The Global Bioresource Center, Australia). Luciferase reporter construct was kindly provided by Dr. Simon Chu (Prince Henry's Institute of Medical Research, Australia). Cells were transfected using Lipofectamine Reagent obtained from Invitrogen (Sydney, Australia).

### 2.2. Plant Material

The stem barks of* Millettia macrophylla* were harvested at the former reserve of Ediki (Kumba, southwest region of Cameroon) in January 2010. Our botanical sample was identified and authenticated by Mr. Victor Nana, botanist at the National Herbarium of Cameroon (HNC) in Yaounde (voucher specimen number 49654/HNC). The well-dried and pulverized stem bark of* M. macrophylla* (2500 g) was macerated in 10 liters of dichloromethane (DCM) for 72 h at room temperature, and 25 g of extract was obtained after filtration and evaporation of the solvent using a rotary evaporator in vacuum. The residue insoluble material was thereafter steeped in 10 liters of methanol for 72 h (MeOH), filtered through Whatman paper N°4, and concentrated to give 53.4 g of methanol extract of* M. macrophylla*. For phytochemical analysis of these extracts, the LC analyses were performed on a Thermo Finnigan HPLC system consisting of a vacuum degasser (Discovery SUPELCO HS C-18 column (25 × 4.6 mm, 5 *μ*m particle size)), a quaternary pump, an autosampler, and a DAD (diode array detector) (Thermo Finnigan, San Jose, CA) as previously reported [18].

### 2.3. Animal Models

Fifty female Wistar rats (*Rattus norvegicus* [19]) aged 3 months (250 g) were obtained from the breeding facility of the Laboratory of Animal Physiology, University of Yaounde 1, Cameroon. They had free access to a standard soy-free rat chow and water* ab libitum*. Animal housing and experiments were carried out following the guidelines of the institutional Ethics Committee of the Cameroon Ministry of Scientific Research and Technological Innovation, which has adopted the guidelines established by the European Union on animal care (CEE Council 86/609).

### 2.4. Study Design

#### 2.4.1. Experiment 1:* In Vitro* Antagonization of* M. macrophylla* Extracts with ICI 182,780

In a previous study, we have found that* M. macrophylla* extracts are able to transactivate the estrogen receptors *α* and *β*, in human embryonic kidney 293T cell line (HEK293T) [18]. In the present study, we investigated whether this transactivation is lost in the presence of a pure antiestrogen ICI 182,780. To accomplish our goal, HEK293T were transiently transfected with the estrogen *α* receptor expression plasmid (200 ng—HEK293T-ER*α* cells) or the estrogen *β* receptor expression plasmid (200 ng—HEK293T-ER *β* cells), together with the double estrogen response element (ERE) fused to a luciferase reporter (250 ng—(ERE)2-tk-Luc) plasmid and *β*-galactosidase reporter plasmid using Lipofectamine Reagent. They were then treated with different concentrations of* M. macrophylla* extracts alone or in combination with ICI 182,780 (500 nM) for 24 h. Cells treated with E2 (10 nM) served as positive control. Luciferase reporter gene assays were performed using a commercial kit (Promega, Australia) according to the manufacturer's instructions. Luciferase activity was measured and normalised against *β*-galactosidase activity determined by using the 2-nitrophenyl *β*-D-galactopyranoside (ONPG) method (Sigma-Aldrich, Sydney, Australia). Each experiment was performed in at least duplicate and repeated three times.

#### 2.4.2. Experiment 2:* In Vivo* Antagonization of* M. macrophylla* Extracts with ICI 182,780

This experiment aimed to elucidate whether the estrogenic activity observed* in vivo* with* M. macrophylla* extracts is mediated through ERs. All substances (E2B, ICI 182,780, and* M. macrophylla* extracts) used in this experiment were dissolved in olive oil. The injection volume was 0.3 mL subcutaneously.

Fifty female Wistar rats were ovariectomized. Fourteen days after endogenous hormonal decline, animals were randomly distributed into 10 groups of 5 rats each. Five animal groups were treated as follows. The negative control received the vehicle (olive oil) and the positive control was treated with estradiol benzoate (E2B) at the dose of 0.75 *μ*g/kg BW/d. Three groups received either DCM extract at the dose of 300 mg/kg or MeOH extract at the doses of 100 and 300 mg/kg BW/d. The last remaining five groups were treated with the same substances and at the same doses combined with ICI 182,780 at the dose of 300 *μ*g/kg BW/d. After the 3-day treatment, animals were sacrificed; the uterine weight, total protein levels in uteri, uterine, and vaginal epithelial heights, and mammary gland were assessed.

### 2.5. Biochemical Analysis

Total uterine protein levels were determined in uteri using colorimetric methods described by Gornall et al. [20].

### 2.6. Histological Analysis

The histomorphological parameters of mammary gland, uteri, and vagina were assessed as described previously [18].

### 2.7. Statistical Analysis

The data from each experimental group were expressed as mean ± SEM. One-way analysis of the variance (ANOVA) followed by Dunnett's test for multiple comparisons and the Student's* t-*test was used for statistical comparison between different control and treated groups for* in vivo* and* in vitro* experiments, respectively. The significance of the difference was fixed at *P* < 0.05.

## 3. Results

### 3.1. *In Vitro* Antagonization of* M. macrophylla* Extracts with ICI 182,780

**Figure 1:**
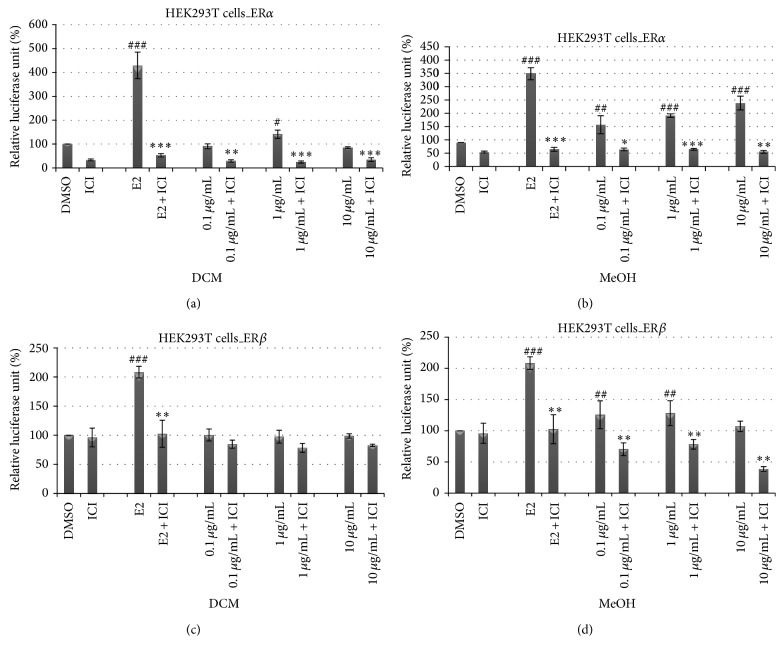
Effects of DCM and MeOH extracts on the activation of estrogen *α* and *β* receptors in HEK293T cells in the absence or in the presence of ICI 182,780 at 500 nM. The effect of DCM and MeOH extracts on estrogen *α* and *β* receptors activity in the transiently transfected HEK293T-ER*α* and HEK293T-ER*β* cells was investigated by measuring reporter gene-coupled luciferase activity. The relative luciferase units (RLU) were measured in the presence of DMSO (0.1%), E2 (10 nM), DCM, and MeOH extracts. ^#^ *P* < 0.05, ^##^ *P* < 0.01, and ^###^ *P* < 0.001 compared with control; ^*^ *P* < 0.05, ^**^ *P* < 0.01, and ^***^ *P* < 0.001 compared with the same dose of each substance without ICI 182,780.

As expected, ICI 182,780 significantly (*P* < 0.001) blocked the transactivation induced by E2 alone in both HEK-ER*α* and HEK-ER*β* (Figure 1). The results obtained after cotreatment of HEK293T cell lines transiently transfected with ER*α* and ER*β* showed a significant inhibition (*P* < 0.001) with* M. macrophylla* extracts and ICI 182,780 (Figure 1). This result confirms that* M. macrophylla* extracts induced their* in vitro* activity through ERs.

### 3.2. *In Vivo* Antagonization of* M. macrophylla* Extracts with ICI 182,780

**Figure 2:**
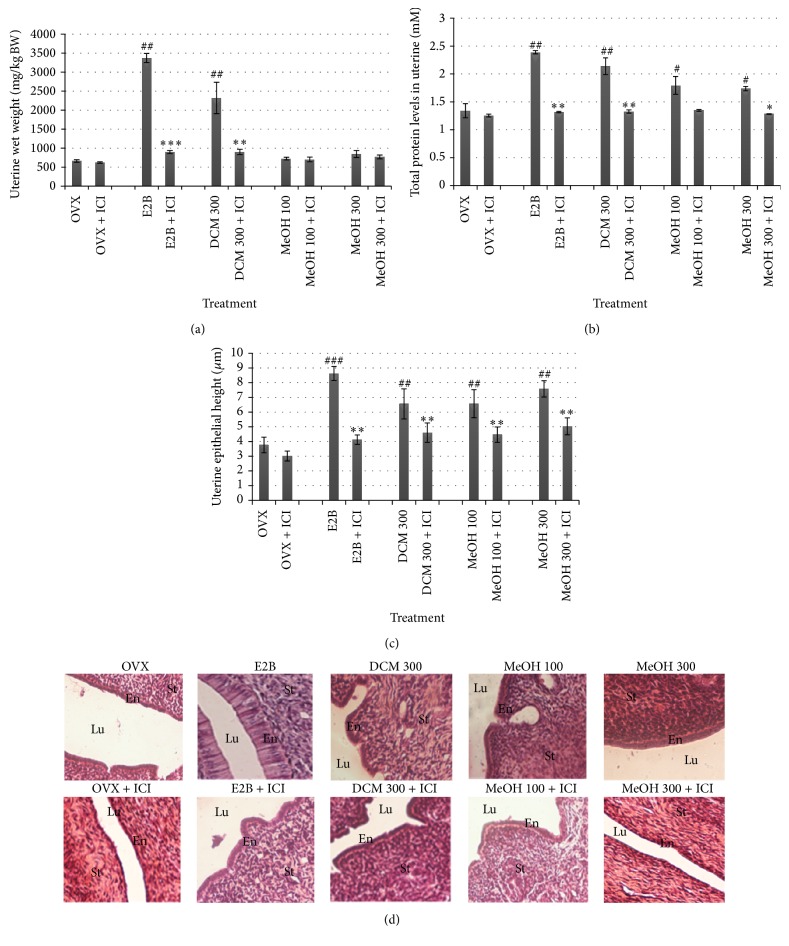
Effects of 3-day treatment with* M. macrophylla* extracts combined or not with ICI 182,780 on the uterine wet weight (a), total protein levels in uterine (b), uterine epithelial height (c), and microphotographs (d). OVX = OVX animals treated with the vehicle (OVX); E2B = OVX animals treated with estradiol benzoate at 0.75 *μ*g/kg BW; DCM = OVX animals treated with the dichloromethane extract of* M. macrophylla*; MeOH = OVX animals treated with the methanol extract of* M. macrophylla*. ^#^ *P* < 0.05, ^##^ *P* < 0.01, and ^###^ *P* < 0.001 compared with control; ^*^ *P* < 0.05, ^**^ *P* < 0.01, and ^***^ *P* < 0.001 compared with the same dose of each substance without ICI 182,780. Lu: lumen; En: Endometrium; St: Stroma.

Regarding the uterine parameters, after the* in vivo* 3-day treatment, antiestrogen ICI 182,780 (Faslodex: 300 *μ*g/kg) totally abrogated (*P* < 0.001) the increase of uterine wet weight induced by E2B (Figure 2(a)), and it significantly (*P* < 0.01) decreased uterine total protein level (Figure 2(b)) and uterine epithelial height (Figures 2(c) and 2(d)). Compared with DCM treated group, the cotreatment of animal with DCM extract and ICI 182,780 induced a significant (*P* < 0.01) decrease in the uterine wet weight. MeOH extract did not increase the uterine wet weight. However, it did significantly (*P* < 0.01) increase total protein levels and uterine epithelial height. ICI 182,780 significantly blocked the increase of these aforementioned parameters when cotreated with DCM and MeOH extracts (Figures 2(b), 2(c), and 2(d)).

**Figure 3:**
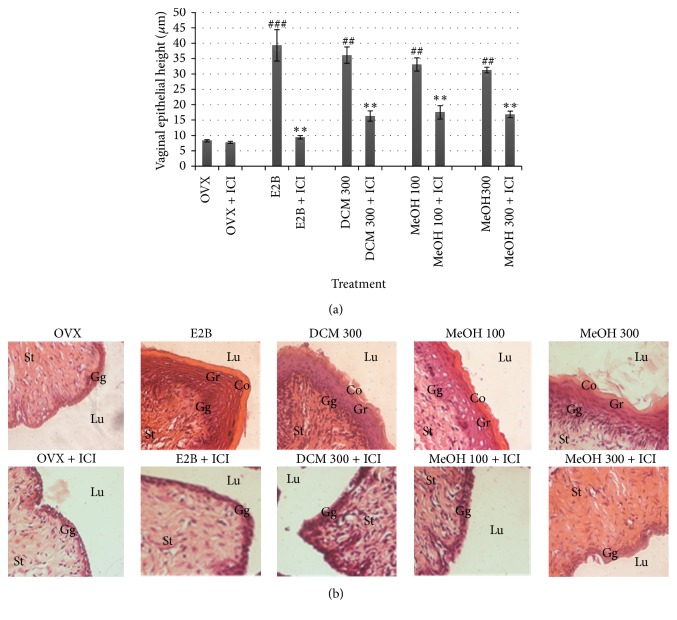
Effects of 3-day treatment with* M. macrophylla* extracts combined or not with ICI 182,780 on the vaginal epithelial height (a) and microphotographs (b). OVX = OVX animals treated with the vehicle (OVX); E2B = OVX animals treated with estradiol benzoate at 0.75 *μ*g/kg BW; DCM = OVX animals treated with the dichloromethane extract of* M. macrophylla*; MeOH = OVX animals treated with the methanol extract of* M. macrophylla*. ^#^ *P* < 0.05, ^##^ *P* < 0.01, and ^###^ *P* < 0.001 compared with control; ^*^ *P* < 0.05, ^**^ *P* < 0.01, and ^***^ *P* < 0.001 compared with the same dose of each substance without ICI 182,780. Lu: lumen; Co: stratum corneum; Gr: stratum granulosum; Ge: stratum germinativum.

As shown in Figure 3(a), E2B treatment significantly (*P* < 0.001) increased the vaginal epithelial height by 266.71% as compared with control (OVX). Treatment with* M. macrophylla* extracts increased the vaginal epithelial height (*P* < 0.01) at all tested doses. Cotreatment of* M. macrophylla* extracts with ICI 182,780 (Faslodex: 300 *μ*g/kg) significantly (*P* < 0.01) abrogated the increase of vaginal epithelial height induced by E2B or* M. macrophylla* extracts treatment alone (Figure 3(a)). These effects were materialized in histological sections by the loss of the presence of stratification (stratum granulosum or stratum corneum) to a thin stratum germinativum consisting of few cell layers as observed in Control group (OVX) (Figure 3(b)).

**Figure 4:**
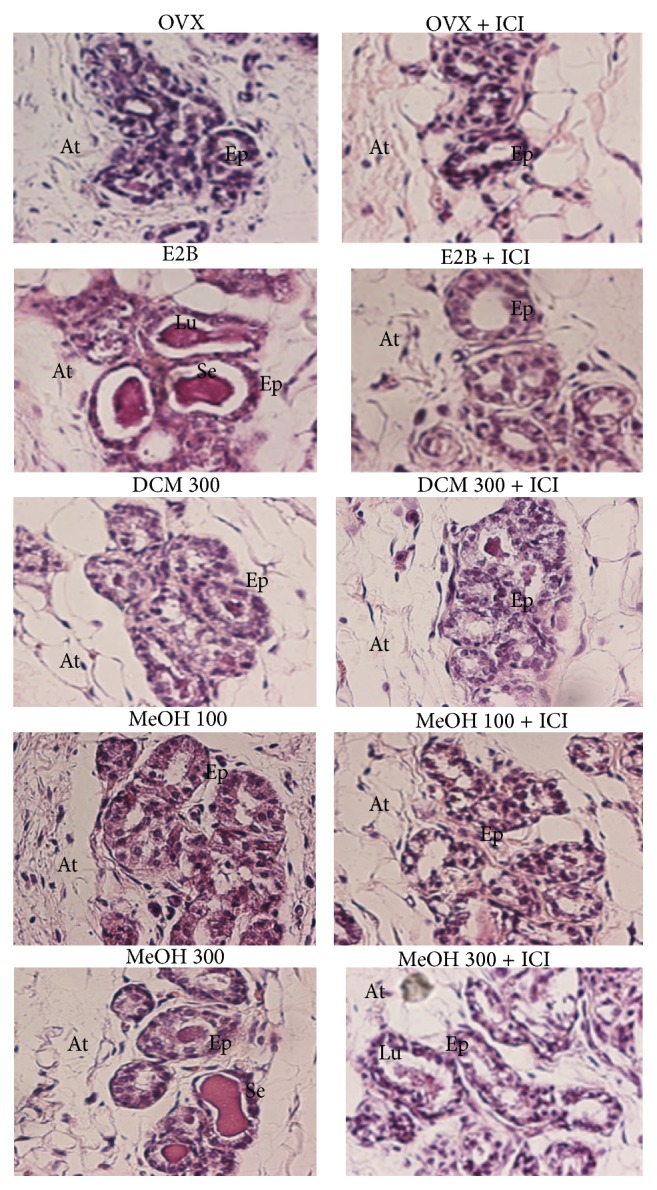
Effects of 3-day treatment with* M. macrophylla* extracts combined or not with ICI 182,780 on mammary gland. OVX = OVX animals treated with the vehicle (OVX); E2B = OVX animals treated with estradiol benzoate at 0.75 *μ*g/kg BW; DCM = OVX animals treated with the dichloromethane extract of* M. macrophylla*; MeOH = OVX animals treated with the methanol extract of* M. macrophylla*. Lu: lumen of alveoli, Ep: alveoli epithelium, At: adipose tissue, Se: eosinophil secretion.

As shown in Figure 4, treatment with E2B induced the increment of the diameter and the lumen of alveoli as well as an abundant eosinophil secretion in the lumen of alveoli. The MeOH extract increased the diameter of alveoli at all tested doses, however it induce eosinophil secretion only at the high dose. DCM extract failed to induce mammotrophic effect. The results of the cotreatments with ICI 182,780 and DCM or MeOH* M. macrophylla* extracts showed that ICI 182,780 inhibits the mammotrophic effects induce by this substance alone; this was materialized by the loss of eosinophil secretion and the increment of acini height.

These results suggest that* M. macrophylla* induced its* in vivo* estrogenic effects through ERs.

## 4. Discussion

*M. macrophylla* has demonstrated pharmacological effects on estrogen target organs of female [18], osteoporosis [21], and male reproductive system of Wistar rats [22]. We hypothesize that* M. macrophylla* might mediate some of its actions by binding to the estrogen receptor, as it shares many estrogen-like actions in various physiological systems. To accomplish our goal, we used ICI 182,780 (Faslodex) which is the most potent estrogen receptor antagonist of the class of 7*α*-alkylamide that has been approved for treatment of postmenopausal breast cancer patients who fail to respond to tamoxifen therapy [23]. It was reported that the ability of agonists of ERs to activate the transactivation ligand-dependent or ligand-independent is completely inhibited by ICI 182,780 [24]. In our study, ICI 182,780 significantly blocked* M. macrophylla* extracts-induced transactivation of both *α* and *β* estrogen receptor subtypes* in vitro*. These results suggest that the induction of transactivation by* M. macrophylla* extracts is due to the binding of its secondary metabolites to ERs, thus corroborating the observation of many authors who also observed the inhibition of ERs (*α* and *β*) transactivation in cellular systems [24–27].

Based on the fact that any beneficial effect obtained* in vitro* is not transferable* in vivo* because of the complexity of this later system, we also antagonized the plant extracts with ICI 182,780* in vivo* in ovariectomized rats. The results showed that cotreatment of the extracts with ICI 182,780 significantly abrogated the increase of uterine wet weight, uterine, and vaginal epithelial height and uterine total protein levels after a 3-day treatment. Moreover, ICI 182,780 inhibits the effect of different treatments (E2B, DCM, and MeOH extracts) on mammary gland. These results corroborate with known ICI 182,780 effects. In fact, ICI 182,780 blocks endometrial proliferation and uterine growth in rats and inhibits growth of breast tumor cells both in cell culture and* in vivo* [25, 28]. ICI 182,780 is known not only for its inhibitory effects on the functional activity of estrogen receptors but also for its ability to reduce the cellular level of ERs [24]. Therefore,* M. Macrophylla* extracts while cotreated with ICI lose their effects probably due to a low level of ERs available to exhibit the estrogen-like effects obtained in the absence of ICI. This confirms that the active principles of* M. macrophylla* bind directly to ERs or at least are part of the regulatory complex at the promoter region of ERs target genes.

Phytoestrogens have been found to bind the estrogen receptor with an affinity 10-fold to 1000-fold lower than estrogen [29].* M. macrophylla* has exhibited estrogen agonistic effects in the range of some well-characterized phytoestrogens; and these estrogenic effects were lost in the presence of ICI 182,780. Therefore,* Millettia macrophylla* should be classified as source of phytoestrogen like a number of plants consumed by humans such as soy, flax, red-clover, hops, citrus, grape, block cohosh, dong quai, ginseng, licorice, and wild Mexican yam [30]. Our results are interesting when considering the data from epidemiological surveys and nutritional intervention studies, indicating that some phytoestrogens ameliorate menopausal symptoms and are protective against a variety of disorders, including cardiovascular disease, cancer, hyperlipidemia, and osteoporosis [31–34].

## 5. Conclusion

The estrogenic activities of* M. macrophylla* extracts may be due to one or more of its constituents that bind to ERs to induce their biological effects. More* in vitro* and* in vivo* studies will be needed to further characterise the molecular mechanism of action that underpins* M. macrophylla* extracts estrogenic activities. Nevertheless,* M. macrophylla* should be classified as a novel source of potent phytoestrogen.
